# Survival Analysis of Risk Factors for Mortality in a Cohort of Patients with Tuberculosis

**DOI:** 10.1155/2020/1654653

**Published:** 2020-09-05

**Authors:** Yi Xie, Jing Han, Weili Yu, Junping Wu, Xue Li, Huaiyong Chen

**Affiliations:** ^1^Department of Prevention, Haihe Hospital, Tianjin University, Tianjin, China; ^2^Tianjin Key Laboratory of Lung Regenerative Medicine, Tianjin, China; ^3^Department of Medical Administration, Haihe Hospital, Tianjin University, Tianjin, China; ^4^Department of Basic Medicine, Tianjin Institute of Respiratory Diseases, Tianjin, China

## Abstract

Identify the treatment effects and risk factors for mortality in patients with pulmonary tuberculosis receiving antituberculosis treatment under the Directly Observed Treatment Short-Course (DOTS) program to reduce the mortality rate of tuberculosis. A retrospective cohort analysis was conducted on the outcomes of antituberculosis treatment of 7,032 patients with tuberculosis in the DOTS program, in the Tuberculosis Management Information System from 2014 to 2017 in Tianjin, China. The Kaplan–Meier method and multifactor Cox proportional risk regression model were used to analyze the risk factors for mortality during antituberculosis treatment under DOTS. The success rate of antituberculosis treatment was 90.24% and the mortality rate was 4.56% among 7,032 cases of tuberculosis in Tianjin. Cox regression analysis showed that advanced age, male sex, human immunodeficiency virus (HIV) positivity, first sputum positivity, retreated tuberculosis, and a delayed visit (≥14 days) were risk factors for mortality in patients with pulmonary tuberculosis receiving antituberculosis treatment under DOTS. The treatment effects in patients with pulmonary tuberculosis during antituberculosis treatment under DOTS were positive in Tianjin. Advanced age, male sex, HIV positivity, first sputum positivity, retreated tuberculosis, and a delayed visit (≥14 days) increased the risk for mortality during antituberculosis treatment.

## 1. Introduction

Tuberculosis (TB) is a chronic respiratory infectious disease caused by *Mycobacterium tuberculosis*. TB is a major public health problem worldwide. The World Health Organization's 2019 Global Tuberculosis Report indicated an estimated 10 million new patients with TB worldwide, an estimated global TB death toll of 1.24 million, and a mortality rate of 16/100,000 individuals in 2018 [1]. TB remains one of the top 10 causes of death worldwide. At present, China has approximately 900,000 new patients with TB annually, ranking 3rd among 30 countries with high TB burdens worldwide [2]. In 2018, the number of TB-related deaths in China was 37,000 and the mortality rate was 2.6/100,000 individuals, ranking 29th among 30 countries with high TB burdens [1]. Since 1992, China has implemented the modern Directly Observed Treatment Short-Course (DOTS) strategy, which has improved the survival rate of patients and reduced the spread and prevalence of TB [3]. However, in the network reporting system of infectious diseases in China, the number of reported TB cases and deaths ranks high in the epidemiology of class A and class B infectious diseases. TB is the third leading cause of death among major infectious diseases and more than 70% of deaths among patients with TB occur in the first 2 months of TB treatment [4, 5]. Therefore, it is important to explore the risk factors of TB death under the DOTS strategy.

TB mortality is an important indicator to evaluate the effectiveness of tuberculosis control and to measure the burden of TB. A TB death-related surveillance system has been established in China. Analyzing and comparing the causes of TB death in information systems is of great significance for selecting the evaluation factors for TB death and predicting epidemic situations of TB deaths. Several studies evaluating the risk factors for death during TB treatment have suggested factors related to age, sex, comorbid conditions, bacteriological status, host immune status, and substance abuse. However, the findings are inconsistent due to differences in the enrolled subjects, the burden of TB in the involved countries, human immunodeficiency virus (HIV) infection prevalence, and socioeconomic status [6–12].

This study retrospectively analyzed the effect of supervision and treatment on newly diagnosed TB patients designated hospitals in Tianjin from 2014 to 2017. The factors influencing the death of TB patients after receiving supervised treatment were analyzed to provide a basis for reducing the mortality of TB.

## 2. Methods

### 2.1. Subjects

This registration-based cohort study collected data on newly diagnosed and registered patients with TB in the outpatient and inpatient departments of Haihe Hospital, a TB-designated hospital in Tianjin, from January 1, 2014, to December 31, 2017. Data from a total of 7,163 patients with pulmonary TB were collected. Pulmonary TB was diagnosed according to the TB Diagnostic Criteria (ws288-2008) and Guidelines for the Diagnosis and Treatment of Tuberculosis by the Chinese Society for Tuberculosis, Chinese Medical Association, combined with patient past history, clinical manifestations, and laboratory examination results. A total of 131 patients who refused anti-TB treatment, died on the day of diagnosis, changed diagnosis, and were lost to follow-up were excluded; thus, 7,032 cases were finally included as research subjects.

### 2.2. Data Sources

The basic information of patients with TB included in the study was collected by querying the *Tuberculosis Information Management System in China* and the hospital information system. In China, there is a compulsory nominative notification of all TB cases directly to the Ministry of Health of the People's Republic of China. The medical staff of Tuberculosis Prevention and Control should register patients diagnosed with TB within 24 hours. The information system mainly includes the following content: sex, age, nationality, occupation, treatment classification (primary and secondary treatment), sputum smear results at the time of diagnosis, time from symptom appearance to diagnosis, time from diagnosis to treatment, presence of pulmonary cavities, human immunodeficiency virus (HIV) test results, drug resistance, treatment plan, anti-TB treatment period, and prognosis of patients with TB. These data did not involve epidemic data or compromise the personal privacy of specific patients with TB.

### 2.3. Treatment Principles

According to the requirements of the “Law of the People's Republic of China on the Prevention and Control of Infectious Diseases” and the Guidelines, DOTS control strategy and follow-up management were implemented by TB prevention and control personnel. The patients were followed up monthly by telephone or home visits. According to the *Guidelines*, the patients regularly visited the designated hospital for follow-up examinations, including sputum smear, sputum culture, chest imaging, blood routine tests, and liver and kidney function assessment. The study result was death during the follow-up period, including all-cause death.

The requirements of the *Guidelines* for the supervision of the treatment period are as follows. (1) Patients with newly diagnosed pulmonary TB are generally supervised for 6 months; if the sputum smear test results of newly smear-positive pulmonary TB patients remained positive at the end of 2 months, the intensive treatment is extended for 1 month and the treatment is supervised for 7 months. (2) Patients with retreated pulmonary TB were generally supervised for 8 months; those who could not use streptomycin were provided 1 month of intensive treatment and 9 months of supervised treatment. If the retreated smear-positive pulmonary TB patients remained positive for sputum bacteria at the end of the second month after treatment, patients treated with the streptomycin regimen should extend the retreatment and intensive treatment for 1 month, with supervised treatment for 9 months, while patients who did not use the streptomycin regimen extend the intensive period of treatment for another month, with the treatment plan unchanged in the continuous period and the supervised treatment for 10 months. The deadline for follow-up was December 31, 2018.

### 2.4. Definitions

- Cure: patients with pulmonary TB confirmed by bacteriology with negative sputum smear or culture results in the last month of treatment and who had been negative at least once before
- Treatment completion: patients who completed the course of treatment and who were negative for sputum bacteria at least once in the course of treatment; however, the sputum bacteria results could not be known due to no sputum examination or sputum in the last month and did not meet the definition of failure
- Failure: patients who remained positive for sputum smear or culture for 5 months or more
- Death: death for any reason during treatment
- Loss of visit: the patient was not treated or the treatment was interrupted for 2 months or more for any reason
- Unable to evaluate: including referral to other TB prevention and treatment institutions or not knowing the treatment outcome
- Treatment success: patients who were cured or who completed treatment

### 2.5. Definitions of Outcome Variables and Survival Time

Initiating event was as follows: the subjects were diagnosed with TB, initiated antituberculosis treatment, and entered the research queue. End event (outcome variable) was as follows: death occurred during the course of the supervised treatment. The survival time from the initiation of antituberculosis treatment to the end of the supervision period was the observation survival time, and the survival time of those who died was the time from the initiation of antituberculosis treatment to death. Truncated data were as follows: for the subjects who did not die during the treatment or at the end of the supervision period, such as those with outcomes of cure, completion of treatment, loss, or discontinuation of adverse reactions, the truncated time was the time when the aforementioned truncated events occurred.

### 2.6. Statistical Analyses

The SPSS19 software was used for statistical analyses of the data. The measurement data are presented as mean ± standard deviation ($\bar{x}+s$) or median and quartile (M (p25–p75)) according to the data distribution. The classification data were assessed using composition ratios, and the chi square test was used for rate comparisons. This study included all patients who met the inclusion criteria and were entered the cohort according to the diagnosis time from 2014 to 2017. Ten variables were analyzed, including age, sex, ethnicity, patient type, first sputum bacteria results, pulmonary cavity, HIV status, drug resistance results, patient delay, and diagnosis delay. The sample size of 7032 with 321 end events is enough according the rule of thumb that Cox models should be used with a minimum of 10 events per predictor variable (EPV) [13, 14].

The survival probability of patients during TB treatment was analyzed using the Kaplan–Meier (KM) method and log-rank tests were used to compare survival curves. A Cox proportional hazards regression model was used to determine factors associated with the risk of death in TB patients. All variables with *P* < 0.2 in the univariate analysis were included in the multivariate Cox regression model. *P* < 0.05 was considered statistically significant. For multicollinearity diagnosis of variables, the tolerance of all independent variables greater than 0.1 and the variance inflation factor (VIF) less than 10 indicate that there was no multicollinearity among independent variables. For the categorical variables, the Kaplan–Meier survival curve method was used to test the hypothesis was met and to determine whether the survival curve was crossed. Intersection of the Kaplan–Meier survival curves of each group of categorical variables indicated that the model hypothesis may not be satisfied. For continuous variables, the residual method was applied and a Schoenfeld residual diagram was drawn for judgment.

The Cox model adopted the forward stepwise regression method based on partial maximum likelihood estimation. The risk function was *h* (*t*) = *h*0 (*t*) exp (*β*1·*X*1 + *β*2·*X*2 + ⋯ + *βm*·*Xm*), where *h*0 (*t*) is the benchmark risk function and *X* and *β* are the observed variables and their regression coefficients, respectively. The inspection level *α* was set to 0.05 (two-sided).

## 3. Results

### 3.1. Subject Characteristics

From January 1, 2014, to December 31, 2017, 7,163 patients with TB were diagnosed and registered in the outpatient and inpatient departments of our hospital. A total of 131 patients (1.82%) were excluded because of treatment refusal, death on the day of diagnosis, or a change in diagnosis. A total of 7,032 patients received standardized antituberculosis treatment and were included in the evaluation of antituberculosis efficacy. Among them, 4,921 (69.98%) were male and 2111 (30.02%) were female; the average age was 50.12 ± 19.99 (range: 12–101) years. There were 5,638 cases (80.18%) of initial TB and 1,394 cases (19.82%) of retreated pulmonary TB. Sputum smear positivity was found in 3,302 cases (46.96%), and sputum smear negativity were found in 3,730 cases (53.04%). A total of 1,476 patients (20.99%) had cavities in the lungs, and 5,556 patients (79.01%) had no lung cavities. Nineteen (0.27%) patients were HIV positive, and 7,013 (99.73%) were HIV negative. The results showed that 902 patients (12.83%) had developed multidrug resistance (MDR) and 6,130 patients (87.17%) were drug sensitive. A total of 321 (4.56%) patients died and 6,711 (95.44%) survived; 50.47% (162/321) of the patients died in the intensive period 2 months of the treatment.

### 3.2. Analysis of the Effect of Standardized Antituberculosis Treatment in the Period of Supervision and Follow-Up

The outcomes of antituberculosis treatment in the 7,032 patients were as follows: 6,346 (90.24%) were successfully treated, 321 (4.56%) died, 40 (0.57%) were lost, 99 (1.41%) failed, 189 (2.69%) developed MDR, 4 (0.06%) discontinued the treatment because of adverse reactions, and 33 (0.47%) had other outcomes. The success rates of antituberculosis treatment in smear-positive and smear-negative patients were 85.43% (2821/3302) and 94.51% (3525/3730), respectively, both of which exceeded 85%, thus achieving the expected treatment goals.

During DOTS treatment, 321 patients died, and the mortality rate was 4.56%. The median age of these 321 patients was 71 (60–81) years, ranging from 16 to 101 years; 263 were males (81.93%), and 58 were females (18.07%). The survival time of patients who died was 1.97 (0.62–4.03) months, and 50% (162/321) of the deaths occurred 2 months of the treatment. The 1-year cumulative survival rate of patients with TB was 94.11%. The 1-year cumulative survival rates of patients with primary and retreated TB were 94.86% and 91.11%, respectively, as shown in Table 1.

### 3.3. Factors Influencing the Mortality during Standardized Antituberculosis Treatment

Univariate Cox analysis was used to analyze 10 factors, including age, sex, nationality, treatment classification, first sputum bacteria results, the presence of pulmonary cavities, HIV results, drug resistance, patient delay (d), and diagnosis delay (d). The results showed that age, sex, treatment classification, results of first sputum bacteria, HIV results, and patient delay were all statistically significant (*P* < 0.05), while ethnicity, the presence of pulmonary cavities, drug resistance, and delay in diagnosis were not (*P* > 0.05), as shown in Table 2.

**Figure 1:**
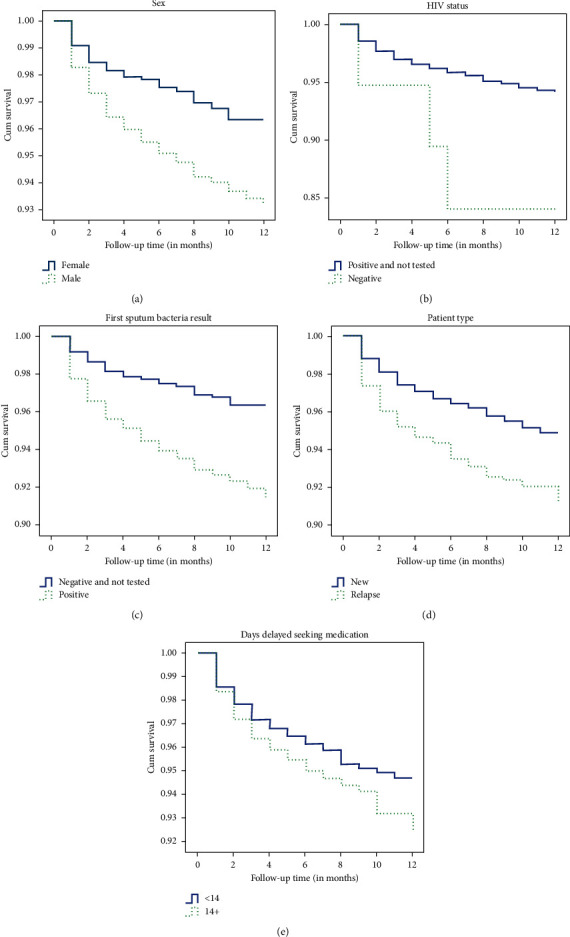
The plot of the estimate of Kaplan–Meier survivor curves of TB patients under DOTS. (a) Sex, (b) HIV status, (c) first sputum bacteria result, (d) TB patient type, and (e) days delayed seeking medication.

**Figure 2:**
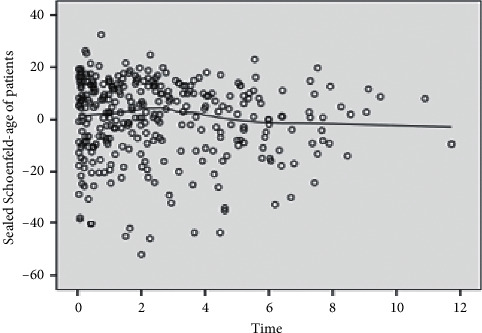
Graphs of the scaled Schoenfeld residuals and their loess smooth curves for the covariates: age of patients.

The survival curve shown in Figures 1(a)–1(e) supports the proportional hazards hypothesis. The Kaplan–Meier survival curves of each group did not cross, indicating that the proportional hazards assumption was met. The plots of the scaled Schoenfeld residual shown in Figure 2 support the assumption of proportional hazards. The figure is random, is smooth, and approximates a horizontal through zero or slope approximately equal to zero.

The multivariate Cox proportional risk model was used to analyze the mortality of patients with TB in the short-term supervision treatment period (0 = no, 1 = death); age, sex, treatment classification, first sputum bacteria result, HIV test result, and patient delay were the independent variables. The results showed that the main risk factors for death during antituberculosis treatment were advanced age, male sex, HIV positivity, first sputum bacteria positivity, retreated TB, and patient delay ≥14 days. The differences were statistically significant (*P* < 0.05), as shown in Table 3. The risk of death increased 1.059 times with each year of increase in age. Advanced age was associated with a higher risk of death. The risk of death was 1.847 times higher for male patients, 3.514 times for patients with HIV positivity, 1.892 times for patients with first sputum bacteria positivity, 1.343 times for patients with retreated TB, and 1.386 times for patients with delayed visit ≥14 days. The calculated tolerance was 0.593–0.998 and the variance inflation factor was 1.002–1.685, indicating the lack of multicollinearity between the variables. The Cox regression equation was as follows: *h* (*t*) = *h*0 (*t*) exp (0.058 × age + 0.613 × male + 1.257 × HIV positive + 0.638 × sputum positive + 0.295 × retreated + 0.327 × patient delay). The survival functions of different variable grouping factors of patients with pulmonary TB are shown in Figures 1(a)–1(e).

## 4. Discussion

In order to effectively treat and manage patients with TB, the International Union against Tuberculosis and Lung Disease introduced the DOTS strategy in the 1970s–1980s [15]. The main evaluation indicators of the effect of TB chemotherapy are generally the negative conversion rate of sputum bacteria at the end of the strengthening period, the cure rate of patients, and the success rate of treatment. The prognosis of patients with TB has mostly been described directly and simply without considering the influence of multiple factors on the DOTS strategy and patients' living conditions. Survival analysis combines the outcomes of the event and follow-up time [16].

Among the 7,032 patients with pulmonary TB who completed DOTS treatment, the success rate of treatment was 90.2%, and the success rates of antituberculosis treatment of patients with smear positivity and smear negativity were 85.4% and 94.5%, respectively. A total of 321 patients died during treatment, with a mortality rate of 4.6%, which was higher than those in Kaili City (2.1%) and Guangzhou (2.8%) [17], but lower than those in Ethiopia (12.71%) [18], India (6%) [19], and Nigeria (16.6%) [20]. The 1-year survival rate was 94.1% after antituberculosis treatment, and the greatest reduction in the cumulative survival rate was observed in the 2 months of antituberculosis treatment. Fifty percent of the deaths occurred at this stage, which is consistent with the results of other studies [5, 21, 22]. It was reported that 20.5% of the deaths occurred in the first 2 months of antituberculosis treatment [22]. A study in northwest Ethiopia showed that 57% of the deaths occur in the intensive phase of TB treatment [5]. The median survival time from the initiation of treatment to death in this study was 1.9 months, which is consistent with the median survival time of 2 months in two studies in Ethiopia [5, 21].

In the multivariate Cox analysis, the mortality risk of male patients was 1.8 times higher than that of female patients (RR = 1.847, 95% CI = 1.387–2.459). This finding is consistent with the results reported by Zenebe and Tefera [23] and Babalik et al. [24]. The higher risk of male deaths is due to the high pressure, increased participation in social activities, high labor intensity, excessive smoking and alcohol consumption, and poor resistance. However, studies have also shown that there is no significant difference in the survival rates between sexes [18]. Age is a risk factor for the survival of patients with TB. The risk of death increases by 5.9% (RR = 1.059, 95% CI = 1.051–1.067) with each year of increase in age, which is consistent with the results of other studies [18, 19, 25]. This finding may be related to low immunity, atypical symptoms, long onset time, more basic diseases, and poor health awareness in elderly patients. Therefore, we should strengthen TB screening in the elderly to achieve early detection, diagnosis, and treatment.

Patient delays refer to more than 14 days between the onset of patients' clinical symptoms and the first visit to a medical institution. Diagnosis delays refer to more than 14 days between patients' first visit to a medical institution and the diagnosis of TB [26]. This study showed that a risk factor for TB-related death was the visit delay, which led to 1.4 times (RR = 1.386, 95% CI = 1.096–1.753). The Cox regression model showed that a delay in diagnosis did not affect the survival of patients with pulmonary TB. This finding is different from those observed in the context of real-life settings and experiences [27]. This may be because TB is a chronic respiratory infectious disease and a delay of diagnosis of more than 14 days did not increase the risk of death in patients in the treatment period. In addition, the medical level of Tianjin is relatively high, especially the application of advanced TB gene diagnosis technology in recent years, which eliminates the fundamental impact of diagnosis delay on mortality in the pulmonary TB treatment period. Early detection and treatment are advocated for TB prevention and treatment to improve the prognosis and quality of life.

Coinfection with HIV promotes the progression and deterioration of TB and leads to rapid death. Many studies have shown that the mortality risk for such patients is high [18, 20, 28]. HIV/TB coinfection can accelerate the reduction in immune resistance and increase the risk of TB incidence and death. Patients with sputum smear positivity not only have a large amount of bacterial discharge and strong infectivity but also have a higher severity. Retreatment of TB is mostly complex and serious, mostly due to irregular or unreasonable chemotherapy. Therefore, it is more difficult to retreat TB than to provide initial treatment, making the mortality rate higher. TB recurrence has become a challenge in the prevention and control of TB in China. The success rate of retreated patients is lower than that of newly treated patients. The low cure rate of retreated TB is the root cause of drug-resistant TB, especially multidrug-resistant TB, which seriously hinders TB prevention and treatment. Therefore, the relevant departments should strengthen TB publicity and education of retreated patients and actively study effective treatment programs for drug-resistant TB.

In the end TB strategy [29], the World Health Organization proposed that the number and incidence rate of TB-related deaths may decrease by 95% and 90%, respectively, in 2035. To achieve this, the government, medical institutions, and TB prevention and control departments need to collaborate to fully implement the DOTS strategy. In China, the DOTS strategy has been fully promoted and achieved some results. However, the relevant departments cannot relax their vigilance and further measures are needed to strengthen the tracking, follow-up, and management of male, elderly, HIV-positive, retreated, and smear-positive TB patients, to further improve the treatment success rate. Focusing on adverse reactions and complications in administering antituberculosis treatment at an early stage will help improve the antiviral treatment effect and reduce the mortality rate [30].

This study performed a retrospective cohort analysis. Due to the limitations of the research methods and economic conditions, the survival analysis did not include nutritional risk status, comorbidities, socioeconomic status, social support, tobacco-alcohol, liver and kidney function, T-SPOT, and GeneXpert data. The outcome variable was all-cause death and death caused by tuberculosis was not distinguished from coincidental death from other causes. A future multicenter prospective cohort study is anticipated that will fully consider the relevant influencing factors to establish a more stable risk model.

## 5. Conclusion

The treatment effects in patients with pulmonary tuberculosis during antituberculosis treatment under DOTS were positive in Tianjin. Advanced age, male sex, HIV positivity, first sputum positivity, retreated tuberculosis, and patient delay (≥14 days) increased the risk for mortality during antituberculosis treatment.

## Figures and Tables

**Table 1:** Survival probabilities throughout the course of treatment in 7,032 tuberculosis patients.

Follow-up period (month)	Number of patients	Death	Censored	Cumulative survival probability (%)
First	7,032	104	83	98.51
Second	6,845	58	48	97.67
Third	6,739	49	58	96.96
Fourth	6,632	27	42	96.57
Fifth	6,563	24	49	96.21
Sixth	6,490	25	157	95.84
Seventh	6,308	13	3530	95.56
Eighth	2,765	13	715	95.05
Ninth	2,037	3	1107	94.85
Tenth	927	3	304	94.49
Eleventh	620	1	124	94.32
Twelfth	495	1	68	94.11

**Table 2:** Univariate analysis of risk factors for death during the treatment period in 7,032 tuberculosis patients.

Variable	Total *N* = 7,032	Death status		HR	95% CI		*P*
		Died *N* = 321 (N, %)	Alive *N* = 6,711 (*N*, %)		Lower	Upper	
Age (years)				1.059	1.052	1.067	<0.001^*∗*^

Sex							
Female	2,111	58 (2.7)	2,053 (97.3)	1.000			
Male	4,921	263 (5.3)	4,658 (94.7)	1.968	1.481	2.615	<0.001^*∗*^

Ethnicity							
Predominant group (Han)	6,920	313 (4.5)	6,607 (95.5)	1.000			
Ethnic groups	112	8 (7.1)	104 (92.9)	1.597	0.792	3.222	0.191

Patient type							
New	5,638	218 (3.9)	5,420 (96.1)	1.000		
Relapse	1,394	103 (7.4)	1,291 (92.6)	1.776	1.400	2.252	<0.001^*∗*^

First sputum bacteria results							
Negative/not tested	3,730	105 (2.8)	3,625 (97.2)	1.000			
Positive	3,302	216 (6.5)	3,086 (93.5)	2.430	1.925	3.068	<0.001^*∗*^

Pulmonary cavity							
No	5,556	248 (4.5)	5,308 (95.5)	1.000			
Yes	1,476	73 (4.9)	1,403 (95.1)	1.137	0.876	1.477	0.334

HIV status							
Negative/not tested	7,013	318 (4.5)	6,695 (95.5)	1.000			
Positive	19	3 (15.8)	16 (84.2)	3.483	1.117	10.858	0.031^*∗*^

Drug resistance results							
Non-drug resistance	6,130	276 (4.5)	5,854 (95.5)	1.000			
Drug resistance	902	45 (5.0)	857 (95.0)	1.259	0.919	1.726	0.152

Patient delay (d)							
<14	5,044	214 (4.2)	4,830 (95.8)	1.000			
≥14	1,988	107 (5.4)	1,881 (94.6)	1.291	1.023	1.628	0.031^*∗*^

Diagnosis delay (d)							
<14	2,696	121 (4.5)	2,575 (95.5)	1.000			
≥14	4,336	200 (4.6)	4,136 (95.4)	1.015	0.810	1.272	0.897

^*∗*^ *P* < 0.05.

**Table 3:** Results of multivariate analysis with a Cox proportional hazard model of risk factors for death during the treatment period in 7,032 tuberculosis patients.

Variable	*β*	S.E.	*Wald* *χ*^2^	*P*	HR	95.0% CI	
						Lower	Upper
Age	0.058	0.004	221.864	0.000	1.059	1.051	1.067

Sex							
Female					1.000		
Male	0.613	0.146	17.639	0.000	1.847	1.387	2.459

HIV							
Negative/not tested					1.000		
Positive	1.257	0.583	4.655	0.031	3.514	1.122	11.009

First sputum bacteria results							
Negative/not tested					1.000		
Positive	0.638	0.120	28.123	0.000	1.892	1.495	2.395

Patient type							
New					1.000		
Relapse	0.295	0.122	5.844	0.016	1.343	1.057	1.706

Patient delay (d)							
<14					1.000		
≥14	0.327	0.120	7.436	0.006	1.386	1.096	1.753

## Data Availability

The data used to support the findings of this study are included within the article.
